# Impact of Gold Nanoparticles and Ionizing Radiation on Whole Chromatin Organization as Detected by Single-Molecule Localization Microscopy

**DOI:** 10.3390/ijms252312843

**Published:** 2024-11-29

**Authors:** Myriam Schäfer, Georg Hildenbrand, Michael Hausmann

**Affiliations:** 1 Kirchhoff-Institute for Physics, Heidelberg University, Im Neuenheimer Feld 227, 69120 Heidelberg, Germany; myriam.schaefer@kip.uni-heidelberg.de (M.S.); georg.hildenbrand@th-ab.de (G.H.); 2 Faculty of Engineering, University of Applied Sciences Aschaffenburg, Würzburger Str. 45, 63743 Aschaffenburg, Germany

**Keywords:** gold nanoparticles, ionizing radiation, single molecule localization microscopy, persistent homology of point patterns, principal components analysis of chromatin organization

## Abstract

In radiation tumor therapy, irradiation, on one hand, should cause cell death to the tumor. On the other hand, the surrounding non-tumor tissue should be maintained unaffected. Therefore, methods of local dose enhancements are highly interesting. Gold nanoparticles, which are preferentially uptaken by very-fast-proliferating tumor cells, may enhance damaging. However, the results in the literature obtained from cell culture and animal tissue experiments are very contradictory, i.e., only some experiments reveal increased cell killing but others do not. Thus, a better understanding of cellular mechanisms is required. Using the breast cancer cell model SkBr3, the effects of gold nanoparticles in combination with ionizing radiation on chromatin network organization were investigated by Single-Molecule Localization Microscopy (SMLM) and applications of mathematical topology calculations (e.g., Persistent Homology, Principal Component Analysis, etc.). The data reveal a dose and nanoparticle dependent re-organization of chromatin, although colony forming assays do not show a significant reduction of cell survival after the application of gold nanoparticles to the cells. In addition, the spatial organization of γH2AX clusters was elucidated, and characteristic changes were obtained depending on dose and gold nanoparticle application. The results indicate a complex response of ALU-related chromatin and heterochromatin organization correlating to ionizing radiation and gold nanoparticle incorporation. Such complex whole chromatin re-organization is usually associated with changes in genome function and supports the hypothesis that, with the application of gold nanoparticles, not only is DNA damage increasing but also the efficiency of DNA repair may be increased. The understanding of complex chromatin responses might help to improve the gold nanoparticle efficiency in radiation treatment.

## 1. Introduction

Besides surgery, radiotherapy with or without chemotherapy or immunotherapy is, so far, the most effective and successful treatment against cancer [1]. Tumors that are successfully treated by photon radiotherapy are often (but not always) fast-growing ones. This is due to the fact that fast-proliferating tumor cells show a higher sensitivity to DNA damage induction than the slowly proliferating healthy cells in the environment. In addition, tumor cells can accumulate genomic alterations and genome instabilities so that drugs and targeting agents can become more effective. This makes DNA further vulnerable to ionizing radiation (IR). The difference in radiosensitivity between the tumor and healthy cells surrounding the tumor defines the therapeutic window in radiotherapy [2,3]. However, this window may be very narrow or not existent at all since tumor cells are either more radioresistant than the environment or compensate for DNA damaging by increased repair or enhanced tolerance to chromatin aberrations [4]. Therefore, precise radiation dose delivery and cell selectivity in radiotherapy are important for an individual treatment strategy and its success.

Besides novel qualities of irradiation techniques like high-LET particle radiation, i.e., protons [5,6] or heavy ions [7,8] with or without application of a FLASH beam [9], another promising approach to further selectively enhance the radiation sensitivity of tumors is provided by the local incorporation of nanoparticles from high-Z materials (Z = atomic number of the element), like gold into the tumor tissue or tumor cells, respectively. This pretreatment can then be followed by irradiation with an adapted dose value [10,11,12,13,14,15].

The original idea underlying this effect of radiosensitization in selected cell types arises from the phenomenon that high-Z materials have a high electron content and, thus, high photoelectric absorption. This leads to the emission of secondary electrons (Compton electrons, Auger electrons) upon irradiation [11,16,17], which are short-ranged. Thus, they induce additional damage within a microscopic or even nanoscopic volume [18,19]. These ejected electrons increase the biological effectiveness of the ionizing radiation exposure due to an increase of DNA damage through dissociative electron attachments [20,21] or water radiolysis and reactive oxygen species (ROS) production [21,22,23].

The main problem of radiosensitization by nanoparticles is their cytoplasmic but not nuclear localization after incorporation. While nanoparticles of sizes up to ~50 nm can cross the cell membrane, they do not cross the nuclear membrane. They do not penetrate into the cell nucleus [10,11,24,25] unless they are modified for instance by DNA and then transferred by a transfection protocol [26,27,28]. It would be adventurous to locate the nanoparticles as close as possible to the membrane of the nucleus [24,27].

The synergistic benefit of the combination of high-Z nanoparticles and ionizing radiation should lead to an enhanced therapeutic window due to more efficient tumor cell killing by locally enhanced radiation and DNA damaging [29]. As mentioned above, healthy cells internalize high-Z nanoparticles less efficiently than tumor cells [30]. This has the consequence that non-tumor cells have a higher opportunity to survive radiation treatment. Or, in other words, the irradiation dose can be decreased to better protect the healthy cells while the tumor cells are killed in the presence of nanoparticles. Radiosensitizing of cells and tissues by high-Z nanoparticles was experimentally confirmed in cell culture and animal models [14,15,27,28,29,30,31,32]. For numerous cell types, this increased cell death was observed after the application of nanoparticles to the cell culture medium several hours prior to radiation exposure [10,33,34]. However, trials to repeat these findings for instance with other cell types or other nanoparticles very often failed [31], although computer simulations based on atomic physics and physico-chemistry [21,35] promised convincing outcomes.

Thus, the biological processes responsible for nanoparticle-mediated (tumor) cell radiosensitization remain elusive and suggest that nanoparticle application could by no means be completely explained by the production of additional DNA-damaging electrons and radiation scattering. It much more verifies our very inconclusive understanding of biological response effects provoked by nanoparticles and radiation exposure.

In this context, it may not be surprising that quantification of nanoparticle-enhanced radiation-damaging effects by colony-forming assays and cell survival curves—the gold standard for determination of biological radiation effects—might not go far enough to completely describe the cell response to nanoparticle-enhanced radiation damaging. Although in some cases after additional treatment of the nanoparticles, slight but significant effects were observed in survival curves [15,27], a majority of results did not show any clear difference between survival curves after radiation exposure with and without nanoparticle application [31]. At first glimpse, this suggests that cells might be unaffected by nanoparticles. However, this outcome raises questions: If nanoparticles are cytotoxic, scatter radiation, and release electrons of different energy spectra, cells should respond in some way. This does not exclude that these responses are complex with antagonistic components. Therefore, a deeper insight into the cell nucleus and potential responses appears to be necessary.

Recently, it has been shown that chromatin is a complex, self-organizing system [36,37,38,39,40,41], and that its 3D organization is characteristic of the cell type [42,43] and its genetic and epigenetic activity. Upregulation and downregulation of genes seem to be correlated with reversible re-organization of heterochromatin [43,44]. For example, due to entropic forces, the broken ends of a chromatin double-strand break induced inside the core of a densely packed heterochromatin block are transported to the heterochromatin border [45,46,47]. Therefore, the surrounding heterochromatin is relaxing [48,49]. After completing the repair, the heterochromatin seems to be re-packed into the original density [49]. In other words, chromatin response to ionizing radiation shows that DNA damage response and repair [50,51,52,53,54,55] is associated with the re-organization of the chromatin as a system as a whole [43], so that, besides damage presentation, a flow of protein activations, i.e., epigenetic processes, may become possible.

This means that defined changes and re-arrangements of spatial chromatin organization reflect potential activities and can be also used as a measure for chromatin response to gold nanoparticles in general [42,43]. Thus, the aim of this study is to find out chromatin-response reactions to gold nanoparticle-enhanced irradiation that occur without impact on cell survival but may open enough opportunities for additional antagonistic regulations. By means of super-resolution Single-Molecule Localization Microscopy (SMLM) and novel mathematical approaches to data evaluation, it will be shown that, although no difference in cell survival occurs for cells irradiated with or without gold nanoparticles, chromatin organization reacts differently. In other words, the article aims to show that unaffected cell survival after gold nanoparticle exposure does not mean that the cells do not react. The cell model SkBr3 [56] irradiated in the presence or absence of gold nanoparticles—known as the most promising anti-tumor radiosensitizers [57]—is well established in breast cancer treatment and radiation research. It was, therefore, chosen for all experiments. We will show that the nano-scaled spatial organization of heterochromatin represented by H3K9me3 methylation sites, or of so-called transposonable chromatin elements represented by ALU sequences [58,59,60,61,62,63,64], are, in general, affected by our treatments in a characteristic way. We also show how γH2AX is spatially organized at DNA damage sites and how this organization changes during repair processes with and without the presence of gold nanoparticles.

## 2. Results

**Figure 1:**
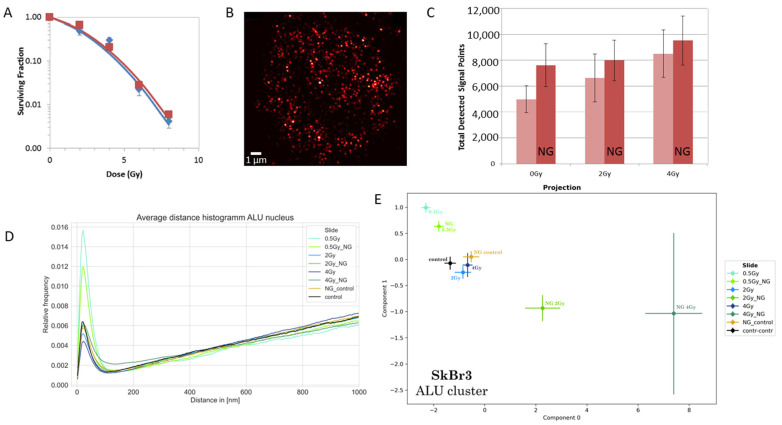
(**A**) Clonogenic survival of SkBr3 cells after irradiation with indicated doses of 6 MV X-rays in the presence and absence of 10 nm gold nanoparticles, respectively. A semi-logarithmic plot of cell survival is shown. The red curve corresponds to SkBr3 cells, treated with gold nanoparticles and irradiated. The blue curve corresponds to SkBr3 cells, irradiated, but not treated with gold nanoparticles. Error bars are the standard deviation of surviving fractions across three replicates. No differences in the survival fractions were found with and without gold nanoparticle incorporation. (Note: This figure was originally published in [31] and is reproduced with permission of The Licensor through PLSclear.) (**B**) SMLM image of a cell nucleus reconstructed from the orte matrix of the ALU labeling sites. The intensity of the points represents the number of next neighbors. (Scale bar: 1 µm.) (**C**) Histogram of the mean numbers of ALU signals detected in cell nuclei for different radiation conditions. Light-red columns represent control cells not treated with gold nanoparticles. Dark-red columns represent cells incubated with gold nanoparticles (“NG”). The error bars show the standard deviation of signal numbers. (**D**) Ripley distance frequency distribution of ALU loci for different treatment conditions. The curves represent the relative frequencies of the pairwise distances of all ALU point loci-measured. (Note: Control cells are those named 0 Gy in (**A**,**C**)). (**E**) PCA of the persistent homology data of the ALU loci in clusters (see peak in (**D**), which shows an increase of short distances below 200 nm). Mean values of component 1 vs. component 0 are shown. In this latent space, “component 0” is the vector in the n-dimensional orthogonal vector space of persistent imaging with the largest variability (variance). Component 1 is a vector orthogonal to component 0 with the second largest variance. The error bars of the components represent the standard deviation. The application of gold nanoparticle leads to a right shift in component 0 if the results for the same doses are compared for specimens with and without gold nanoparticle incorporation. With harsher treatment, the variation between the individual nuclei is increasing.

SkBr3 cells were prepared with and without gold nanoparticles and irradiated with different doses of ionizing radiation. As described in detail elsewhere [31], the cells were subjected to a colony-forming assay in order to obtain quantitative data on cell survival after radiation exposure. No significant difference was found for the survival fractions of the irradiated cells treated with gold nanoparticles in comparison to the control cells irradiated but without gold nanoparticle incorporation (Figure 1A).

Since it was shown that cell types can be categorized by topological calculations of the point patterns of ALU labeling sites [43] obtained by COMBO-FISH [64,65], which also re-arrange in a characteristic way during DNA repair after ionizing radiation exposure [42], the SkBr3 cells were subjected to ALU labeling, and the loci of the labeling points were measured by SMLM and subjected to SMLM dataset analyses. In Figure 1B, a typical image is shown as it can be obtained from the orte-matrix. In all cases, the average number of ALU labeling points counted in the middle plane of the cell nuclei showed a tendency towards higher values for cells with gold nanoparticles incorporated (Figure 1C). Although this increase was not significant in the statistical sense, it indicated that the application of gold nanoparticles had an impact on chromatin organization. Since the number of ALU sites is fixed in the human genome, i.e., they cannot increase, the increased number of labels could be explained by improved accessibility for the COMBO-FISH probes to the ALU sites, which can be interpreted as the result of an increased chromatin relaxation.

Looking at the Ripley statistics (Figure 1D), in all cases, the distances above about 200 nm follow a nearly random distribution. The clustering of ALU labeling points was only visible at lower distances. However, any systematic difference between cell nuclei with and without gold nanoparticle incorporation was not observed. Therefore, the topology of the point patterns within clusters (defined by a minimum of 30 points within 200 nm cluster size) was investigated. After the application of PCA (Figure 1E) to the results of persistent homology/imaging, a significant shift of component 0 to the right was observed for all gold nanoparticle specimens (irradiated and control ones) in comparison to the specimens without gold nanoparticles. This shift was considerably higher (indicating a considerable change in chromatin organization) than the shift between specimens irradiated with 2 Gy and 4 Gy but without gold nanoparticles. In addition, the standard deviation increased in both components (0, 1) and was especially large for higher doses (≥2 Gy) in comparison to the control or the lower-dose exposure experiments (500 mGy).

All these findings indicated that, although cell survival was not influenced by gold nanoparticle incorporation in comparison to irradiation only, there was a clear impact on chromatin organization by the application of gold nanoparticles, and the treatment changed the chromatin organization in a characteristic way that was found in all single cells analyzed.

**Figure 2:**
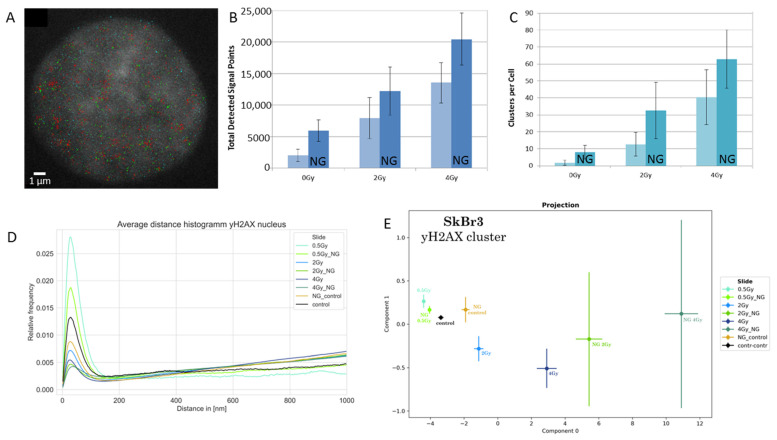
(**A**) Example of a merged image of γH2AX clusters (green) within the ALU point pattern (blue; see also Figure 1B). The cell nucleus was treated with gold nanoparticles and then irradiated with 2 Gy. Areas of overlap between γH2AX and ALU are indicated in red. Scale bar: 1 µm. (**B**) Histogram of the mean numbers of γH2AX signal points detected in cell nuclei for different treatment conditions. The blue columns represent the mean numbers of γH2AX signals in cells detected 30 min after irradiation. The light-blue columns are the mean numbers of signal points in cells not treated with gold nanoparticles. The dark-blue columns are the mean numbers of signal points from cells incubated with gold nanoparticles (“NG”). Error bars show the standard deviation of signal numbers. (Note: This figure was originally published in [31] and is reproduced with permission of The Licensor through PLSclear.) (**C**) Histogram of the mean numbers of γH2AX clusters detected in cell nuclei for different radiation conditions. The light-blue columns represent control cells not treated with gold nanoparticles. The dark blue columns represent cells incubated with gold nanoparticles (“NG”). The error bars show the standard deviation of signal numbers. (Note: This figure was originally published in [31] and is reproduced with permission of The Licensor through PLSclear.) (**D**) Ripley distance frequency distributions of γH2AX points for different treatment conditions. The curves show the relative frequencies of the pairwise distances of the detected points. (Note: Control cells are those named 0 Gy in (**A**,**C**).) In addition, treatment conditions of 0.5 Gy are shown, which were not included in the survival experiments of Figure 1A. (**E**) PCA of the persistent homology data of the γH2AX loci in cluster (see peak in (**D**)). Mean values of component 1 vs. component 0 are shown. In this latent space, “component 0” is the vector in the n-dimensional orthogonal vector space of persistent imaging with the largest variability (variance). Component 1 is a vector orthogonal to component 0, with the second-largest variance. The error bars of the components represent the standard deviation. The application of gold nanoparticles leads to a right shift in component 0 if the data of the same irradiation dose with and without nanogold application are compared. With harsher treatment, the variation between the individual nuclei increases, as indicated by the increasing standard deviations.

In the next step, γH2AX labeling was analyzed. γH2AX occurs around DNA double-strand breaks [66,67] and forms typical clusters as shown in Figure 2A. The number of γH2AX labeling points (Figure 2B), as well as the number of clusters (Figure 2C), increased after the application of gold nanoparticles in comparison to the control or irradiated specimens only. Without radiation exposure, the amount of double-strand breaks seemed to be higher, which may be true due to the formation of ROS by gold nanoparticles [23]. This additive effect of gold nanoparticles was also visible for the irradiated specimens.

At first glimpse, the result of Figure 2C seemed to contradict the Ripley curves (Figure 2D) where the relative frequency of clusters (peak below 200 nm) was reduced with increasing dose and with gold nanoparticle application. However, it has to be considered that this is a relative frequency distribution related to all experiments together. With an increase in the harshness of the treatment, the size of the clusters represented by the peak was reduced, while the amount of higher distance values was increased. In other words, the larger clusters relaxed, and small cores of the clusters remained. Such behavior is compatible with the progression of repair.

The PCA of the topology (persistent homology/imaging) of the γH2AX clusters also indicated a strong impact on chromatin organization around double-strand breaks by the treatments applied. γH2AX clusters in specimens treated with gold nanoparticles showed a significant shift to right in the component 0 in comparison to the specimens exposed to the same radiation dose but with the incorporation of gold nanoparticles. With the reduction of the γH2AX cluster size, the variation, especially for component 1, increased, indicating increased topological fluctuations with increased harshness of treatment.

The results described above revealed, on one hand, an increased damaging by gold nanoparticle-supported radiation treatment, as represented by γH2AX 30 min after irradiation. However, this damaging did not influence cell survival at later time points. On the other hand, chromatin organization, as represented by the ALU labeling pattern, showed treatment-dependent modifications, which were compatible with chromatin relaxation 30 min after radiation exposure. This could be due to the heterochromatin relaxation that usually occurs after double-strand break induction in heterochromatin [49]. Relaxed chromatin allows for a better diffusion of proteins in the cell nucleus and, thus, could be a hint for an improved accessibility of repair proteins to damage sides. These findings are supported by the data of γH2AX clusters that indicate progress in repair.

To study chromatin organization changes and damage-induced relaxation of heterochromatin, the methylation sites were labeled with anti-H3K9me3 antibodies. The cells were subjected to the following irradiation scheme, which was applied with and without incorporation of gold nanoparticles: (a) radiation exposure of 2 Gy in two fractions, i.e., irradiation with 1 Gy followed by 30 min cell culturing without treatment and another exposure to 1 Gy again; (b) radiation exposure of 2 Gy in one fraction. In both cases, the cell nuclei were analyzed 30 min after the last radiation treatment. The time gap of 30 min between the 1 Gy radiation fractions was chosen in order to obtain, on one hand, a reasonable repair process (non-homologous end joining is a fast repair process within the first 30 min after radiation exposure). On the other hand, the complete repair process should not have been finished. Details of these experiments were described elsewhere [15].

**Figure 3:**
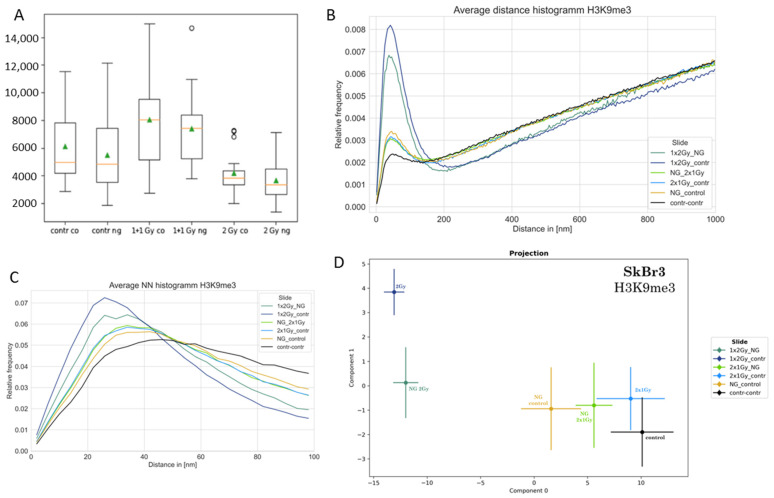
(**A**) Absolute number of fluorescent points detected per image (z-section of 500 nm) for the different experiments (contr = non-irradiated control; 1 + 1 Gy = radiation scheme 1 Gy—30 min—1 Gy; ng = gold nanoparticles; co = control without gold nanoparticles; for further details see text). Each detected point represents a fluorescent antibody against the H3K9 methylation site of heterochromatin. Blinking events within the localization precision were registered as one point. The boxplots show the mean point number of the detected nuclei (green triangle), the median (orange line), the lower and upper quantile (box), and the value range within +/−2 standard deviations (black line). The black circles refer to values that differ more than 1 box length from the median (Note: This figure and a slightly modified figure legend were originally published under CC BY license in [15].) (**B**) Ripley distance frequency distributions of H3K9me3 points (H3K9 methylation sites) for different treatment conditions. (**C**) Histogram of the relative frequencies of the next neighbors (NN) of each H3K9me3 locus. (Note for (**B**,**C**): contr = irradiated control in comparison to the same experiment with gold nanoparticles (NG); 2× 1 Gy = radiation scheme 1 Gy—30 min—1 Gy; NG control = cells with gold nanoparticles but without radiation treatment; contr-contr = control cells without gold nanoparticles and without radiation exposure.) (**D**) PCA of the persistent homology data of the H3K9me3 loci. Mean values of component 1 vs. component 0 are shown. In this latent space, “component 0” is the vector in the n-dimensional orthogonal vector space of persistent imaging with the largest variability (variance). Component 1 is a vector orthogonal to component 0 with the second-largest variance. The error bars of the components represent the standard deviation. The radiation scheme 1 Gy—30 min—1 Gy has no significant difference to the respective control, while the application of gold nanoparticles in general leads to shifts in component 0.

Signal counting of the H3K9me3 tags (Figure 3A) revealed a high variation in all cases, especially because the cells were not synchronized. No statistically significant differences in counts were detected for the different radiation experiments compared to the non-irradiated control. The counts for the 2 Gy in one fraction experiments were slightly lower and less variable compared to the 2 Gy in two fraction experiments and the controls. The correlating experiments with and without gold nanoparticles especially showed the same signal number distributions.

The Ripley distance frequency curves (Figure 3B) revealed a random distribution of the heterochromatin labels in all cases for distances above 200 nm. Below 200 nm cluster formation was represented by the respective peaks. The data showed broad peaks, especially for the 2 Gy in one-fraction samples (with and without gold nanoparticles). This was also accompanied by the shortest next-neighbor distances as shown in Figure 3C. This was compatible with heterochromatin cluster relaxation and more antibody binding due to better chromatin accessibility. Relative to these broad peaks of the 2 Gy in one-fraction experiments, the peaks for the 2 Gy in two-fraction experiments and the gold nanoparticle control experiment without irradiation had about the same width but were considerably lower. This indicated a reduction in the number of such clusters compatible with heterochromatin relaxation in general. Nevertheless, in comparison to the non-treated control (SkBr3 cells have a low clustering of heterochromatin in general [56]), the formation of heterochromatin clusters showed a strong re-organization during the different treatments, which was also shown by the differences in the next-neighbor frequency curves (Figure 3C).

PCA of the topological data analysis (persistent homology/imaging) revealed significant changes in the specimens with gold nanoparticle incorporation compared to the non-treated control in component 0. There was also a significant difference between the experiments of 2 Gy in one fraction and 2 Gy in two fractions in components 0 and 1. However, the 2 Gy in two-fraction experiments did not significantly differ from their correlating controls, which was also compatible with a progressed repair process. No significant difference was also observed between the experiment with and without gold nanoparticles. These results were compatible with chromatin strand repair activities in parallel to the damaging scheme.

**Figure 4:**
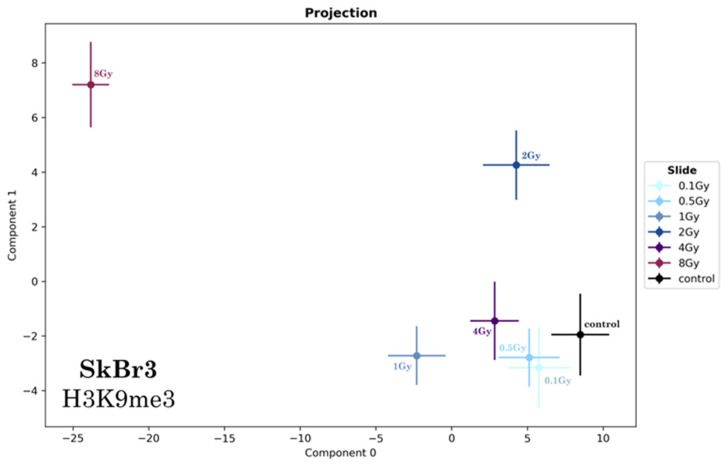
PCA of the persistent homology data of the H3K9me3 loci 30 min after the application of radiation of different doses (no gold nanoparticle application). Mean values of component 1 vs. component 0 are shown. In this latent space “component 0” is the vector in the n-dimensional orthogonal vector space of persistent imaging with the largest variability (variance). Component 1 is a vector orthogonal to component 0 with the second-largest variance. The error bars represent the standard deviation.

In order to support this statement, PCA of heterochromatin topology was executed for specimens 10 min after irradiation of SkBr3 cells with different doses (Figure 4). Here, a clear separation of the 1 Gy and 2 Gy value from the control was observed. Only for irradiation with low doses (<500 mGy), a slight but not significant separation from the control value was found.

**Figure 5:**
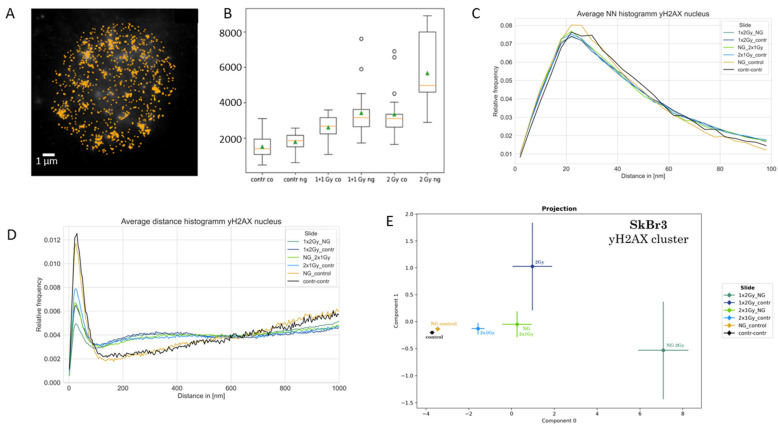
(**A**) Typical example of an artificial image obtained from data of the orte matrix for γH2AX signals 30 min after treatment of SkBr3 cells with gold nanoparticles and 2 Gy radiation exposure. This image is merged with the real widefield DAPI image (shadowed). (Note: This figure and a slightly modified figure legend were originally published under CC BY license in [15]). (**B**) Absolute number of fluorescent points detected per image (z-section of 500 nm) for the different experiments; (contr = non-irradiated control; 1 + 1 Gy = radiation scheme 1 Gy—30 min—1 Gy; ng = gold nanoparticles; co = control without gold nanoparticles; for further details see text). Each detected point represents a fluorescent antibody against γH2AX. Blinking events within the localization precision were registered as one point. The boxplots show the mean point number of the detected nuclei (green triangle), the median (orange line), the lower and upper quantile (box), and the value range within +/−2 standard deviations (black line). The black circles refer to values that differ more than 1 box length from the median (Note: This figure and a slightly modified figure legend were originally published under CC BY license in [15]). (**C**) Histogram of the relative frequencies of the next neighbors (NN) of each γH2AX locus. (**D**) Ripley distance frequency distributions of γH2AX points for different treatment conditions. The curves show the relative frequencies of the pairwise distances of the points. (**E**) PCA of the persistent homology data of the γH2AX loci in cluster (see peak in (**D**)). Mean values of component 1 vs. component 0 are shown. In this latent space, “component 0” is the vector in the n-dimensional orthogonal vector space of persistent imaging with the largest variability (variance). Component 1 is a vector orthogonal to component 0 with the second-largest variance. The error bars of the components represent the standard deviation. Depending on the treatment, not only the size of the γH2AX clusters is changing but also the internal topological arrangement, which results in the different component values.

The distribution of γH2AX labels was also investigated for the 2 Gy experiments described above. In Figure 5A, a representative image of a cell nucleus is shown, which was prepared from the orte matrix. The typical clustering of γH2AX labels is visualized. For better segmentation of the cell nucleus, the image of the γH2AX points was merged with a real widefield DAPI image recorded simultaneously for the same cell nucleus. The number of γH2AX labels was determined 30 min after the last irradiation treatment (Figure 5B). The irradiated cells without gold nanoparticles showed a slightly increased number of γH2AX labels in comparison to the non-irradiated control cells. However, the two different 2 Gy irradiation schemes did not differ in the labeling frequency. The incorporation of gold nanoparticles did not significantly change the number of recorded labels, with the exception of the 2 Gy in one fraction with gold nanoparticles, which showed a highly significant increase.

The frequency distribution of the next-neighbor distances (Figure 5C) was highly similar for all six different treatment cases and had a maximum of about 25 nm. The results of Ripley’s distance frequency curves (Figure 5D) were about the same for the non-irradiated control specimens independent of whether gold nanoparticles were incorporated or not. The cluster peak below 100 nm was continued by a random point distance distribution for larger distance values. For the irradiated samples, the size of the γH2AX clusters did not change significantly. Here, the cluster formation was associated with an increase of the cluster density, which was shown by the second broader peak at about 300 nm distances.

Although a lot of geometrical similarities were found for γH2AX clustering, PCA of the topological analyses revealed a strong shift of component 0 for the specimens with gold nanoparticles incorporated. This is compatible with Figure 2E, where the same behavior was observed for other irradiation conditions. This also indicated that repair processes in cases of gold nanoparticle incorporation could run in a different way, as compared to specimens irradiated without gold nanoparticle enhancement.

## 3. Discussion

In radiotherapy, a successful application of the cell-killing radiation dose to the tumor is required. Therefore, the surrounding non-tumorous healthy tissue should be less or even not affected. Such a dose range is described by the therapeutic window [2]. Unfortunately, the therapeutic window is often very low or does not really exist [3]. Therefore, local dose-enhancement procedures within the tumor cells only would be a considerable breakthrough towards better radiotherapy [29].

High-Z nanoparticles like gold nanoparticles [57] preferentially incorporated into fast-proliferating tumor cells were promising such a breakthrough because of their scattering and absorption behavior of ionizing radiation, which results in the additional release of short-ranged electrons of different energies and increased ROS production [18,19,20,21,23]. Although many experiments done with cell and tissue cultures or in animal models revealed a successful local increase of damaging and cell killing within tumors [32], this breakthrough was extenuated by many contradictory experiments [14,33].

It is assumed that the type of radiation used in combination with high-Z nanoparticles may cause such contradictions. MV radiation sources are frequently used in radiotherapy so that the cell response with gold nanoparticles under such a radiation scheme may be reasonable. However, the absorption of kV radiation by high-Z nanoparticles, especially gold nanoparticles, is known to be more effective than the absorption of MV radiation. So, it might not be expected that the application of MV radiation in combination with high-Z nanoparticles would have a significant effect on cell survival. However, Chithrani et al. [68] showed a dose-enhancement factor of 1.17 for 6 MV x-rays, in comparison to 1.43 for 220 kV and 1.66 for 105 kV x-rays in HeLa cells. Also, Burger et al. [27] reported a significant effect on cell survival after gold nanoparticle application and 6 MV radiation exposure of HeLa-cells.

Therefore, further investigations for understanding the cellular response to high-Z nanoparticle incorporation, as well as the combination of high-Z nanoparticle incorporation and ionizing radiation exposure, are required on a multi-scale approach, starting from single-molecule interactions to whole-cell nucleus responses [21]. For instance, the production of ROS (radical oxygen species) caused by water radiolysis would not only induce additional DNA damage but could also change the distribution of charges and electric potentials in a local environment. This might have an additional impact on the chromatin organization and, thus, radiosensitivity. Also, short-ranged Auger electrons may either cause secondary effects in the cytosol or, depending on the positioning and type of the nanoparticles, induce damage in the heterochromatin near the nuclear membrane. The question of how short-ranged Auger electrons damage chromatin and induce repair processes was investigated in detail and will be the subject of another publication (manuscript in preparation).

In this article, we focused our results on an established model in breast cancer treatment research, the cell line SkBr3. Although other cell lines might have shown different results in detail, we showed all possible effects with one cell line only, since it was not the aim of the study to work out differences between cell lines. Here, the aim of the article was to show exemplary along one cell line that characteristic gold nanoparticle-induced changes in the chromatin organization occur without having an impact on cell survival. In future experiments, it could be investigated whether and how different cell lines may react differently or not and whether this might have some correlations to radiosensitivity. For the first time, it was shown that changes in chromatin organization on the nanoscale after exposure to ionizing radiation of different doses and incorporation of gold nanoparticles can be measured. How such changes depend on the type of nanoparticles (size, material) and on the concentrations of the nanoparticles applied will be the subject of future systematic investigations.

Another aspect for future investigations may be systematic variations of fractionated radiation treatments and different recovery times. It is known that radiosensitivity or radioresistance can be influenced by fractionated treatments under appropriate treatment and recovery time schemes. Understanding the mechanisms behind this might improve applications of nanoparticles in future medical treatments.

On the basis of survival curves obtained by colony-forming experiments that did not show any difference in cell survival after irradiation with or without gold nanoparticle incorporation [14,27,31], it has been hypothesized in this article that the nanoparticle enhanced damaging of chromatin in cell nuclei should be accompanied by another antagonistic effect. Such an antagonistic effect might be an increased repair activity. A small hint towards this direction may be found in Figure 2D. The peaks of the Ripley pairwise distance frequency histograms are always lower for the experiments with gold nanoparticles compared to the experiments with the same dose without gold nanoparticles. Under the assumption that γH2AX clusters are mostly equally sized [69] (supported by the next-neighbor distributions in Figure 5C), such results may reveal that 30 min after irradiation, the number of γH2AX clusters is reduced in cell nuclei of cells containing gold nanoparticles during irradiation. On the other hand, ALU cluster formation is also reduced in experiments of the same dose with gold nanoparticles compared to those without. On first glimpse, assuming that relaxation of chromatin is correlated to DNA damaging, these results would contradict the γH2AX results. However, it has to be considered that all results were obtained 30 min after irradiation. This means that the repair is not completely finished. Assuming that chromatin compaction is the last step of the repair process, ALU might indicate a relaxed chromatin conformation, although the repair process has made considerable progress. To verify such speculative conclusions would require further systematic experiments.

In general, it may be a question of discussion to which extent the detection of ALU-specific COMBO-FISH probes might be advantageous for studies of changes in chromatin organization. The oligonucleotide probes only label a 17mer of the whole and usually flexible ALU region (associated with euchromatin) by one fluorochrome. After DNA double-strand break induction, for instance, the relaxation of this chromatin region is usually small. Also, in the results shown here, the shifts of ALU signal distances (represented by the broadening of the peaks in Figure 1D) are relatively small. This is in agreement with Weidner et al. [42], who found small changes in the next-neighbor distances of ALU signals during the repair process after DNA damage induction by ionizing radiation. However, these small distance shifts seemed to have changed the topology of the ALU signals considerably by reversibility.

In order to better understand whether such small effects are really significant, additional control experiments might be useful. For example, a demethylase could be included, which should reduce DNA methylation, potentially leading to chromatin relaxation and an increase in ALU spacing. Additionally, using a histone acetyltransferase or deacetylase could provide evidence of chromatin condensation or relaxation. Comparing treated samples to untreated or differently treated ones could be an approach to validate that the changes in ALU sequence spacing correlate with chromatin topology modifications. This might strongly suggest that the observed small-distance changes are due to radiation and nanoparticle-induced chromatin distortion. The use of such controls will be applied in future experiments for a better understanding of the findings of chromatin topology changes as a whole system.

It has been assumed that the cell nucleus is a self-organizing system [36,37,38,39,40,41,43,70,71] and, for instance, ALU regions or heterochromatin form cell-type characteristic networks [41,43]. These networks react to any treatment not only by a single molecular response but even more as a system as a whole that could be completely reorganized especially on the nanoscale [42]. Although the results contain a comprehensive dataset that clearly supports these general assumptions, the outcome of cell reactions measured here cannot, per se, be generalized for all cell types and types of high-Z nanoparticles. The verification of such a generalization would require analyses of a broad spectrum of cell types and high-Z nanoparticles. Nevertheless, the example shown here can be seen as proof of the principle that it may be feasible to show different cellular chromatin organization reactions. The present outcome remains basic research to improve our knowledge. However, a better understanding of chromatin response to nanoparticle application offers a way to obtain cell parameters that may be modified to improve cell-killing effects of nanoparticles.

SMLM datasets of point patterns obtained by precise localization of single-labeling molecules for whole chromatin conformation (COMBO-FISH probes for the ALU consensus regions, anti-H3K9me3 antibodies) or γH2AX clusters (anti-γH2AX antibodies) around chromatin double-strand breaks were used and subjected to well-established mathematical procedure and algorithms for point-pattern analyses [42]. With this novel combination of a sophisticated form of super-resolution light microscopy and rigorous mathematics, new insights into the spatial organization of chromatin were obtained.

Although chromatin damage response and chromatin strand repair require complex protein machinery, the focus of this article was drawn on the spatial organization of chromatin, and epigenetic studies were omitted. These may be tasks for future investigations.

Chromatin in cell nuclei is organized in three dimensions. The SMLM datasets used are only 2D and were obtained from an optical section of about 500 nm thickness through the middle plain of the cell nucleus. This might cause the question of to which extent the 2D datasets were representative of the 3D organization of chromatin.

If image processing were to be applied to the SMLM datasets, the reduction down to two dimensions would have also to be considered carefully. In the approach presented here, the datasets were abstracted to coordinate matrices on which mathematical procedures of statistics and topology were applied for parametrizing features of the chromatin in a latent space. Mathematical topologies as persistent homology are scale and rotation invariant [72]. This, on one hand, overcomes the practical problem that each cell nucleus is recorded under another perspective due to random fixation of the nuclei on the slides. On the other hand, topology analyses in combination with PCA reduced the parameter space just to two components (0 and 1) of the highest variances so that, finally, these two values represent the characteristic whole system response neglecting small organization changes (“biological noise”). With other words, based on two values in the latent space only, the main characteristic changes of the system response can be determined.

Although changes in the two values of the latent space indicate changes in the chromatin organization, it cannot always explicitly be reconstructed which change in the 3D-chromatin conformation and geometry occurred at which point in the cell nucleus. Nevertheless, this abstract reductive process applied here has made it possible for the first time to quantify radiation and gold nanoparticle-induced changes of chromatin as a whole.

For the model cell system used here, the application of gold nanoparticles in combination with or without ionizing radiation at different doses revealed changes in the chromatin organization in general and the organization of chromatin damage sites. Such changes were caused by the presentation of chromatin damages to the repair machinery, which, in addition, would have an impact on molecular diffusion flows and chromatin accessibility for proteins. Thus, these spatial organization changes offer new perspectives on the application of gold nanoparticles. With these novel approaches to spatial analysis of chromatin and other novel approaches to epigenetic analyses, a plethora of tools are available for future investigations that might bring answers to, so far, unsolved questions of nanoparticle-enhanced radiation treatment.

## 4. Materials and Methods

### 4.1. Cell Culture, Gold Nanoparticle Uptake, and Irradiation

The human breast cancer cell line SkBr3 was derived from a human adenocarcinoma of the mammary gland tissue (American Type Culture Collection, Manassas, VA, USA) [65]. A detailed description of cell culturing can be found in [65]. In brief: According to the ATCC protocol, the cells were grown in McCoy’s medium supplemented with 10% FBS (Fetal Bovine Serum) without any antibiotics at 37 °C with 5% CO_2_. After 3–4 days, the cells were taken from the culture and reseeded 1:10 into culture flasks. Upon reaching appropriate cell density (approximately 70–90% visible confluence of cells adhered to the bottom of the flask), cells were trypsinized, counted, and diluted to obtain 10^4^ cells/mL. Two milliliters of this homogenous suspension were distributed into each well of a six-well culture plate and incubated overnight, allowing cells to adhere to clean coverslips at the bottom of each well. After removing the medium, the cells were washed in PBS and re-suspended in 2.5 mL of fresh McCoy’s medium (containing no FBS) per well. In each well, 8 μL suspension of commercially available, colloidal, and uncoated gold nanoparticles (10 nm diameter, concentration 5 × 10^12^ nanoparticls/mL; commercially available under: Aurion GoldSol 10 nm; cat. No. 410.011, AURION Immuno Gold Reagents & Accessories, Aurion, Wageningen, The Netherlands) in 2.5 mL of FBS-free McCoy’s medium was added. Untreated control cells received medium only. The cells were incubated at 37 °C for 18 h. After incubation for 18 h and washing with PBS, McCoy’s medium with 10% FBS was added for further cultivation.

The nanoparticles were freshly purchased and tested against aggregates by visual microscopic inspection before application and after adding to the cells. The gold nanoparticles used in the experiments here were not fluorescent in order to avoid fluorescence crosstalk with the antibody labeling of the specimens. So, the uptake of gold nanoparticles could not be counted directly. So, the incorporation of the gold nanoparticles was tested by an alternative check based on the assumption that antibodies binding internally to the membrane are hindered by the gold nanoparticles (for details and results, see Supplementary Materials with Figures S1 and S2, Tables S1 and S2). Furthermore, according to Moser et al. [24], who used fluorescing gold nanoparticles in the same concentration, an uptake of about 9000–10,000 gold nanoparticles could be expected per optical section of about 500 nm through the middle plain of a cell, which is usually analyzed in our SMLM data acquisition procedure. Counting the number of gold nanoparticles in electron microscopy sections and estimating the uptake by the cells used here supported the results of Moser et al. [24], as well as for the SkBr3 specimens prepared here.

Finally, the cells were irradiated at room temperature by 6 MV X-Rays (dose: 0.5 Gy up to 8 Gy) delivered from a linear accelerator used in clinics [27] without a flattening filter (LINAC, Synergy, Elekta AB, Stockholm, Sweden; dose rate 6.67 Gy/min). During irradiation, the surface of the media was about 100 cm distant from the radiation source. In order to avoid effects of beam in-homogeneities on cell irradiation, the local dose within the field of specimen irradiation was measured.

Following irradiation, the cells were again incubated at 37 °C for the specific repair time (mostly 30 min if not mentioned otherwise). This time window of 30 min is reasonable since large chromatin changes become measurable while the formation of γH2AX clusters at the damaged site reaches an optimum.

The medium was removed and the cells were washed with PBS for 5 min at room temperature. Finally, the cells were fixed in 3.7% formaldehyde (freshly prepared from paraformaldehyde) for 30 min at room temperature and washed three times with PBS. For further processing, the specimens were stored in PBS containing 0.05% sodium acid.

### 4.2. Clonogenic Assay (Colony-Forming Assay)

The clonogenic assay was, in principle, adapted from Burger et al. [27] and is described in detail elsewhere [31]. In brief: As described above, SkBr3 cells were grown in six-well plates for 18 h. The medium was removed and the cells were washed with PBS. After incubation in 1 mL 3× Trypsin/EDTA per well at 37 °C for 5 min, the cells were resuspended in 2 mL of McCoy’s medium with 10% FBS. From each well, the cells were transferred to two 15 mL tubes, one for control and the other for treatment with gold nanoparticles. The number of cells was counted with a hemo-cytometer under a light microscope.

The content of each 15 mL tube was diluted to a particular cell number in 400 μL and transferred into single micro-centrifuge tubes, each containing 400 μL of liquid. Five tubes contained cells with gold nanoparticles and five tubes contained cells without gold nanoparticles, one for each radiation dose applied. All 10 tubes were irradiated by 6 MV x-rays delivered from a linear accelerator (LINAC, Synergy, Elekta AB, Stockholm, Sweden; dose rate 6.67 Gy/min) at room temperature. During irradiation, the tubes were positioned about 100 cm distant from the radiation source.

After irradiation, the cells were thoroughly re-suspended to ensure homogeneity of the suspension. One hundred microliters of each tube were transferred into culture flasks so that each flask received about one-quarter of the cells. The cells were incubated in 5 mL of McCoy’s medium with 10% FBS at 37 °C with 5% CO_2_ for 14 days to allow sufficient colony growth. Then, the cells were washed with PBS and fixed with 3.7% formaldehyde (prepared from paraformaldehyde), followed by 70% ethanol fixation for 10 min each.

For visualization of colonies, the cells were stained with Coomassie dye for 40 s and Giemsa solution for 40 min. The colonies, of at least 50 cells, were counted microscopically.

### 4.3. Immunostaing and COMBO-FISH Labeling

Cells were grown on coverslips in six-well plates until 40% confluence. The cells were permeabilized with 0.2% Triton-X100 in 1× PBS for 3 min, washed three times in 1× PBS for 5 min each, and blocked with 2% BSA (bovine serum albumin) in 1× PBS. Then the cells were labeled with rabbit anti-H3K9me3 antibodies (1:400, ab8898 Abcam, Cambridge, UK) for heterochromatin and with mouse anti-γH2AX antibodies (clone JBW301; 1:250, cat. No: 05-636-I, Merck Chemicals, Darmstadt, Germany) at 37 °C for 30 min. After washing three times in PCTG (PBS, 0.1% Casein, 0.05% Tween20 and 0.5% fish gelatin (Sigma–Aldrich, Waltham, MA, USA)) for 5 min each, these primary antibodies were labeled with fluorescent secondary goat anti-rabbit Alexa Fluor^®^488 (ab150077, Abcam, Cambridge, UK) and goat anti-mouse Alexa Fluor^®^568 (Abcam) antibodies for 45 min at 37 °C, followed by three washes in PCTG for 5 min each. The specimens were counterstained with DAPI and embedded in ProLong Gold anti-fade solution (Thermo Fisher Scientific, Waltham, MA, USA) for further microscopic analysis.

In some other cases, immunostaining of γH2AX was performed on treated cells together with a special staining technique for ALU sequences called COMBO-FISH (combinatorial oligonucleotide fluorescence in situ hybridization) [64,65] using a protocol that combines the two staining techniques, immunostaining, and fluorescence in situ hybridization of oligonucleotides. This chromatin-organization preserving method of COMBO-FISH specifically labels ALU sequences throughout the nucleus by hybridization with a 17 bases long complimentary oligonucleotide carrying a fluorescent dye molecule (Alexa Fluor^®^568). In contrast to typical FISH protocols [73] that require thermal denaturation of target DNA into single strands that rearrange chromatin organization, COMBO-FISH maintains the chromatin organization by avoiding high-temperature denaturation of target DNA [64].

While COMBO-FISH protocols are available in detail elsewhere [73], the applied combination protocol [64,65] is briefly described in the following: Coverslips with fixed, treated, and irradiated cells were removed from 4 °C storage and washed with 1× PBS + Mg/Ca to remove residual sodium acid. The cells were submerged in 2 mL permeabilization solution for 3 min at room temperature. Then, they were washed three times with 1× PBS + Mg/Ca for 5 min each before being incubated in blocking solution for 30 min. Cells were then incubated in contact with 100 μL of primary anti-γH2AX antibody solution for 18 h at 4 °C in a humidified chamber. The cells were washed three times with 1× PBS + Mg/Ca for 5 min each, and then again incubated with 100 μL of secondary goat anti-mouse Alexa Fluor^®^647 (Abcam; ab150115) antibody solution for 30 min in a 37 °C humidified chamber. Cells were then washed three times with 1× PBS + Mg/Ca for 5 min before being fixed in 2% formaldehyde (prepared from paraformaldehyd) at 37 °C for 10 min. Post-fixation, cells were again washed three times with 1× PBS + Mg/Ca for 5 min each and incubated in 2 mL 0.1 M HCl for 10 min at room temperature. Cells were then washed three times in 0.05% Triton-X (in 1× PBS + Mg/Ca) for 5 min each on the shaker. Coverslips were then soaked in 2× SSC, and the cells were allowed to equilibrate for 5 min. Following equilibration, cells were submerged in 50% formamide in 2× SSC for 30 min at room temperature to softly denature DNA strands. Each coverslip was then placed cell-side down onto 20 μL of COMBO-FISH probe solution against a stretch of the ALU-consensus region (Alexa Fluor^®^568 TAATCCCAGCACTTTGG: (IBA life science, Goettingen, Germany)) on a microscope slide, and the edges were sealed with fixogum (Marabu, Tamm, Germany). Cells were incubated for 21 h in a 37 °C humidified chamber.

Following incubation, fixogum was removed, and the cells were washed three times in 2× SSC at 37 °C for 10 min each. Coverslips were then soaked in 1× PBS + Mg/Ca, and the cells were allowed to equilibrate for 5 min. Cells were then incubated in contact with 100 μL of DAPI solution for 5 min at room temperature in darkness. After DAPI counter-staining, coverslips were again washed with 1× PBS + Mg/Ca before being placed cellside down onto 20 μL of ProlongGold on a microscope slide. Coverslips were sealed on slides and stored at 4 °C.

### 4.4. Single-Molecule Localization Microscopy (SMLM) and Data Evaluation

SMLM is an advanced technique of super-resolution fluorescence microscopy that makes use of stochastic blinking processes of fluorescent labeling molecules and precise localization (typically in the 10-nanometer range) of these blinking molecules. The fundamental concept of SMLM super-resolution [74,75,76,77,78,79] is based on optical isolation of fluorescent molecules. The dye molecules were excited for fluorescence. However, they can stochastically switch between two different spectral states, the on- (=fluorescing) and off- (non-fluorescing) state (also called “reversible photo-bleaching”), after illumination with a high-power laser. This fluorophore “blinking” allows for spatial separation of individual fluorescent signals. The loci of these blinking events are precisely localized by the barycenter (center of the fluorescent intensity peak) of the Airy disc (=diffraction disk) visualized by the objective lens [79]. The setup for SMLM used in the experiments here is described in detail elsewhere [48,79]. It has an oil-immersion objective lens (100×/NA 1.46) and four lasers (405 nm/491 nm/561 nm/642 nm) with maximal laser powers of 120 mW/200 mW/200 mW/140 mW, respectively. The illumination laser light path is equipped with a LightHub—laser combiner (Omicron Laserprodukte GmbH, Rodgau- Dudenhofen, Germany) and a polychromatic AOTF (AA Opto Electronic, Orsay Cedex, France). The laser beams are shaped by a variable beam expander 10BE03-2-8 (Standa Ltd., Vilnius, Lithuania) and a Flat-Top-Profile-forming optics—PiShaper (AdlOptica GmbH, Berlin, Germany). The fluorescence light is separated from the illumination light by two quadband interference filter glasses F73-410 and F72-866 (AHF Analysentechnik AG, Tübingen, Germany). The blinking events of the labelling molecules are detected by an Andor Ultra EMCCD (iXonUltra 897, Andor Technology, Belfast, Northern Ireland).

Typically, 20–50 cells were recorded for each treatment. The individual cell nuclei were selected by uniform shape as visualized by DAPI staining (using the 405 nm laser). Up to 2000 image frames were acquired at each illumination wavelength used with an exposure time of 100 ms per image. Super-resolution signal coordinates were calculated using in-house software as described elsewhere [42,80].

In brief: The positions of these molecules were calculated according to an algorithm subtracting the brightness values of two successive image frames. Dark states over more than two successive frames were also registered. The barycenter calculation for each recorded point is described in [80]. After conversion from the pixel space into the coordinate space, all molecule loci are registered in the so-called “orte-matrix” that consists of nine columns: (a) the amplitude of the signal in photoelectrons, (b) the lateral y-coordinate in nm, (c) the lateral x-coordinate in nm, (d, e) the measurement errors for x and y coordinates, (f, g) the standard deviations, (h) the number of photoelectrons in the signal i.e., counts, and (i) the number of the image frame in which the signal is found. Signals with too-low intensities are sorted out by a threshold.

**Figure 6:**
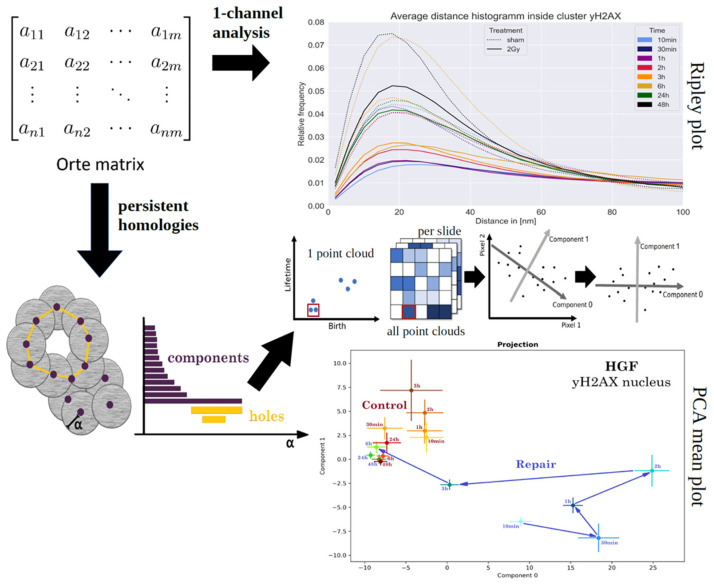
Schematic representation of the different evaluation steps of SMLM datasets. After acquisition of a time series of image frames, the coordinates and other values of all the blinking events of a cell nucleus were integrated into a matrix, the so-called “orte-matrix” (top-left). In the one-channel analysis, the coordinate values of the points were subjected to Ripley statistics. For the representative graph (top right), the point-to-point distances were calculated and represented in a normalized distance frequency histogram. In the lower part, the processes of persistent homology, persistent imaging, and principal component analysis are shown. The point pattern is transferred into a bar code description of components and holes. The lengths of the bars (difference of the α values of the end and the beginning of a particular bar) provide the lifetime; the beginning provides the birth in the one-point cloud. The one-point clouds for all cell nuclei that are considered for evaluation are transferred in pixel images (persistent imaging). Each pixel is compared for all images. The results of these comparisons span an n-dimensional orthogonal vector space. The variations of the pixel values determine the components of the principal component analysis. Finally, the outcome for the components (orthogonal vector values) with the largest variation and the second largest variation determines the latent space (graph bottom right) (for further details, see text in Material and Methods).

The localization data of the labeling molecules are further processed with house-made programs written in Python [42]: The coordinate values of the points were subjected to Ripley statistics [81], a form of spatial statistics that involves stochastic point processes, sampling, smoothing, and interpolation of regional (areal unit) and lattice (gridded) point patterns, as well as the geometric interpretations of the statistical outcome. Using algorithms for Ripley statistics, the point-to-point distances were calculated and represented in a normalized distance frequency histogram from which the geometry of point arrangements can be obtained. A homogeneous point-to-point distance distribution leads to a linear curve with a different slope. The formation of clusters means that smaller distances are more frequent, resulting in a peak (see Figure 6: “1-channel analysis”).

For topological analysis [72], persistent homology [82,83,84] analysis, persistent imaging [85], and principal component analysis [86] were applied as summarized and described in detail in [42] and schematically shown in Figure 6. A major principle of topological analysis is the determination of properties of structures, which are described by the pointillist pattern here. Mathematically, this corresponds to transformations in the topological space defined by the structures. In the experiments on chromatin analyses by SMLM, the attention will be focused on two quantifiable parameters: the number of components (=number of points with which the analysis starts) and holes (=closed configurations like meshes of a net that occur and disappear during the mathematical evaluation process). These elements are independent of each other. In algebraic topology, these elements are also called zero-dimensional and one-dimensional simplicial complexes.

By persistent homology, significant structures of a point pattern are, therefore, obtained by the following process: Each point (=component = simplicial complex of dimension 0) registered in the orte-matrix is surrounded by a virtual circle with an increasing radius. Each component is represented by a bar that starts at 0 (=the given point) and ends at the radius value where two circles attach. In this way, two components merge into one. During this process, enlarged components can form a virtual closed loop (in its simplest form a triangle) with free space inside not covered by the increasing virtual circles. The area of this free space is considered as a hole (simplicial complex of dimension 1). At this moment, another bar starts. When the hole is completely covered by the increasing virtual circles, this bar also ends. With connecting components and, thus, reducing their number, the number of holes is first increasing and then decreasing because more and more holes are closed. By this process, the point pattern of fluorescent molecules obtained by SMLM is transferred into a bar code pattern of two different types of bars: one for the components and one for the holes.

For each cell, the results of the barcodes are transferred into a diagram of “bar lifetime” versus “bar birth”, which is then transferred into a pixel scan (persistent image) in which the pixel intensity correlates to the number of points in a particular pixel of this diagram. These persistent images are obtained for each cell of an experiment. Afterwards, the persistent images are subjected to principal component analysis (PCA). This means that the values of the 1st, the 2nd, the 3rd, the 4th, the nth pixel, etc., of all individual persistent images are separately compared and represented in an n-dimensional orthogonal vector space. The dimension (i.e., the respective vector of this n-dimensional vector space) with the largest variances then forms the component 0 in the final diagram, the latent space (see Figure 6, right bottom). Component 1 is a dimension (vector) perpendicular to component 0 and shows the second large variance of values. In this way, the results of a complex point pattern can be reduced to one value (±standard deviation) of a diagram (latent space) of two dimensions. This means that only two principal components describe the main features that represent the main variances, while small variations (“biological noise”; other orthogonal vectors with fewer variations of values) are neglected. In this way, changes of chromatin organization can be described by shifts of the outcome in the latent space.

## Data Availability

Data are part of the KIP SMLM data archive and can be obtained upon request from the corresponding author.

## Supplementary Materials

The following supporting information can be downloaded at: https://www.mdpi.com/article/10.3390/ijms252312843/s1. References [87,88] are cited in the Supplementary Materials.
