# Supplementary material for: Impact of Gold Nanoparticles and Ionizing Radiation on Whole Chromatin Organization as Detected by Single-Molecule Localization Microscopy
Source: Int J Mol Sci. 2024 Nov 29;25(23):12843. doi: 10.3390/ijms252312843 (PMC11641692; doi:10.3390/ijms252312843)
Supplement: Supplementary file 1 [file ijms-25-12843-s001.zip › ijms-3287567-supplementary.pdf]

Supplementary Material:

Since the gold nanoparticles used in the experiments here did not fluoresce, the potential incorporation was verified by an indirect effect. In [1] the distribution of gold nanoparticles in the cytosol shows that these particles can also be found near the cell membrane inside the cell. This has motivated to label a transmembrane protein with an intra-cellular and an extra-cellular antibody simultaneously and to compare the signal numbers in cells with and without gold nanoparticles.

Table S1: Overview over the experiments and staining

Experiment	Excitation 488 nm	Excitation 568 nm	Gold Nanoparticles
1 (one color)		Her2-kinase	No
2 (one color)		Her2-kinase	Yes
3 (bi-color)	Her2-kinase	Her2-extra cellular	No
4 (bi-color)	Her2-kinase	Her2-extra cellular	yes

For this purpose, the kinase and the extracellular domain of the Her2 protein (further “kinase” and “extra”) were stained in the plasma membrane by appropriate antibodies. Usually these receptors occur at regular distances in the cell membrane. Table S1 shows an overview of the preparations made. Figure S1 shows the numbers of kinase and extra events detected by SMLM. In all cases, the average number of detected signals decreased after the application of gold nanoparticles. In Figure S1, the results of kinase signal reduction is significant compared to the reduction of extra. Table S2 shows the p-values for the null hypothesis, i.e. the probability that two random samples originate from sets of the same mean value. For kinase, the number of signals detected decreases more strongly with the addition of gold nanoparticles than for the more distant extra.

(a) (b)

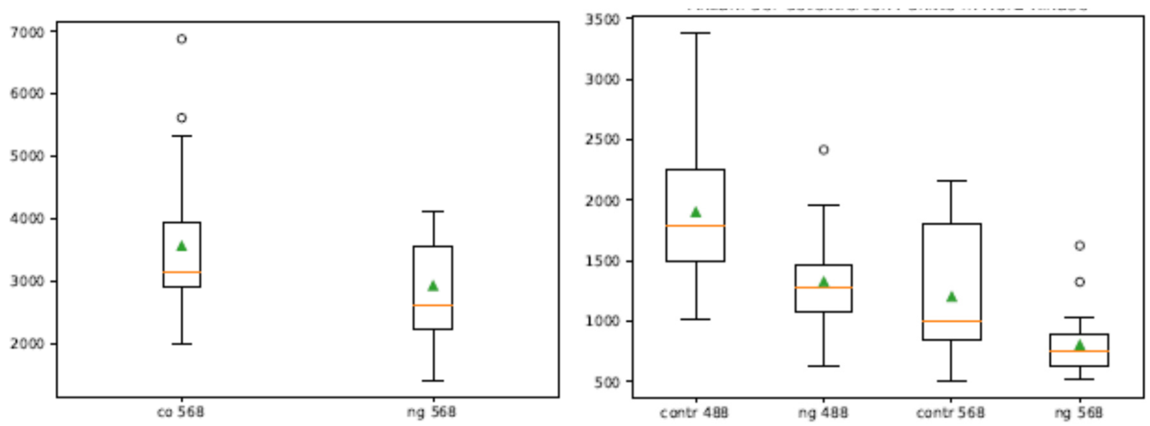

Figure S1: Boxplots of the numbers of detected events. a) for experiment 1 and 2 (Her2 extracellular); b) for experiment 3 and 4 (Her2 kinase and Her2 extracellular)

Table S2: Significance p-values for the reduction of fluorescence (co = control; ng = gold nanoparticles)

Experiment	Excitation 488 nm	Excitation 568 nm	p-Value
1 and 2		kinase co vs. kinase ng	0.001
3 and 4	kinase co vs. kinase ng		0.005
3 and 4		Extra co vs. extra ng	0.058

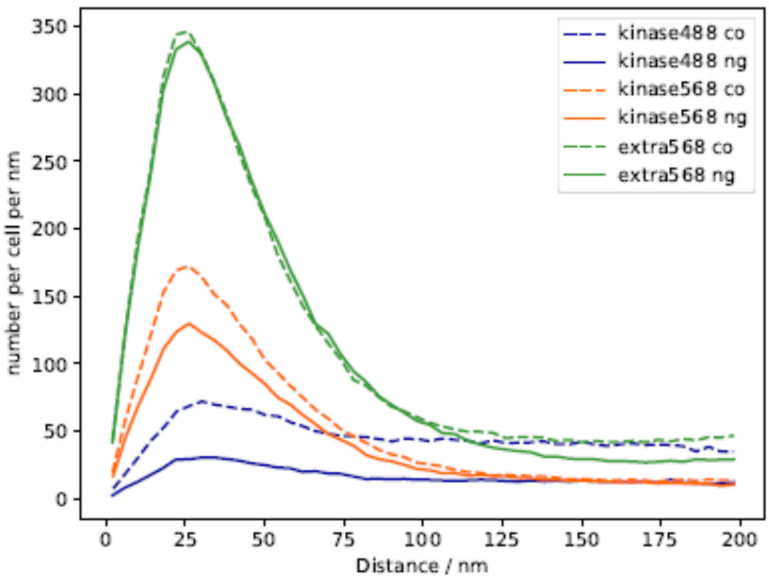

Figure S2: Ripley distance frequency distributions for the different experiments indicate a significant reduction of small kinase distances for those specimens where gold nanoparticles were incorporated into the cells, while the extra signal distances were not affected.

A Ripley distance frequency analysis of the signals was also performed (Figure S2). The distance curves of the extra signals coincide for the cells with and without gold nanoparticles. In contrast, the number of distances below 100 nm for kinase decreased after the addition of gold nanoparticles. This indicates that the number of detected signals decreased significantly when gold nanoparticles were expected at the intramembrane side.

Thus, it can be concluded that the gold nanoparticles either reduced the binding of the intramembrane antibodies or weakened the fluorescence signals to such an extent that they werel below the detection limit. This is consistent with the theory that gold nanoparticles can absorb a photon emitted by a fluorophore and thus reduce the apparent number of fluorophores in the close vicinity [2]. This effect occurs at small distances (< 20 nm). In particular, it was shown that the signal number of Her2 kinase (kinase) decreased significantly more than that of Her2 extracellular (extra). From this it can be concluded that gold nanoparticles were present in the cytoplasm.

References

1. Hausmann, M.; Pilarczyk, G.; Maus, E.; Hesser, J.; Hildenbrand, G.; Super-resolution microscopy of nanogold labeling. In *Nanoparticle Enhanced Radiation Therapy: Principles, Methods and Applications*; Sajo, E., Zygmanski, P., Eds.; IOP Publishing: Bristol, UK, 2020; ISBN 978-0-7503-2396-3.
2. Anger, P.; Bharadwaj, P.; Novotny, L. Enhancement and quenching of single molecule fluorescence. *Phys. Rev. Lett.*, **2006,** *96,* 113002.
